# Supplementary material for: Quantifying the interfacial triboelectricity in inorganic-organic composite mechanoluminescent materials
Source: Nat Commun. 2024 Mar 26;15:2673. doi: 10.1038/s41467-024-46900-w (PMC10966096; doi:10.1038/s41467-024-46900-w)
Supplement: Supplementary file 1 — Supplementary Information [file 41467_2024_46900_MOESM1_ESM.pdf]

Supplementary Information

Quantifying the interfacial triboelectricity in inorganic-organic composite mechanoluminescent materials

Xin Pan[1,2], Yixi Zhuang[2,3,*], Wei He[2], Cunjian Lin[4], Lefu Mei[1], Changjian Chen[2], Hao Xue[2], Zhigang Sun[5], Chunfeng Wang[6], Dengfeng Peng[7], Yanqing Zheng[5], Caofeng Pan[8], Lixin Wang[9,*], and Rong-Jun Xie[2,3,10*]

[1]School of Materials Sciences and Technology, China University of Geosciences Beijing, Beijing 100083, China.

[2]College of Materials, Xiamen University, Xiamen 361005, China.

[3]Fujian Key Laboratory of Surface and Interface Engineering for High Performance Materials, Xiamen University, Xiamen 361005, China.

[4]Graduate School of Advanced Science and Technology, Japan Advanced Institute of Science and Technology, Nomi 923-1292, Japan.

[5]School of Materials Science and Chemical Engineering, Ningbo University, Ningbo 315211, China.

[6]College of Materials Science and Engineering, Shenzhen University, Shenzhen 518060, China.

[7]College of Physics and Optoelectronic Engineering, Shenzhen University, Shenzhen 518060, China.

[8]Beijing Institute of Nanoenergy and Nanosystems, Chinese Academy of Sciences, Beijing 100140, China.

[9]Department of Vascular Surgery, Zhongshan Hospital, Fudan University, Shanghai 200032, China.

[10]State Key Laboratory of Physical Chemistry of Solid Surfaces, Xiamen 361005, China.

[*]Correspondence and requests for materials should be addressed to Y.Z. (email: zhuangyixi@xmu.edu.cn), L.W. (email: wang.lixin@zs-hospital.sh.cn) or R.X. (email: rjxie@xmu.edu.cn).

Table of Contents

Supplementary Fig. 1 SEM images and photographs of the BPC phosphors and ceramics.

Supplementary Fig. 2 Variation in the triboelectric charge (in nC) between different ceramics and organic film.

Supplementary Fig. 3 Variation in the voltage (in V) between different ceramics and organic film.

Supplementary Fig. 4 Crystal structure of the apatite phosphors *M*P*X* (*M* = Ca/Sr/Ba, *X* = Cl).

Supplementary Fig. 5 Distribution of particle size of the *M*P*X* phosphors (*M* = Ca/Sr/Ba, *X* = Cl) after sintering.

Supplementary Fig. 6 Time-resolved ML measurements of samples upon mechanical stimulus.

Supplementary Fig. 7 PL, CL and ML spectra of *M*P*X* phosphors (*M* = Ca, Sr, Ba; *X* = Cl).

Supplementary Fig. 8 Comparison of the ML intensity in BPC@PDMS with and without light exposure.

Supplementary Fig. 9 CL spectra of the *M*P*X* phosphors (*M* = Ca, Sr, Ba; *X* = Cl) by using different accelerating voltages.

Supplementary Fig. 10 TECD of $SrAl_2O_4$:Eu and PDMS film by using the measurement system given.

Supplementary Fig. 11 ML photographs of the SPC@PDMS film under stretching for 2000 times.

Supplementary Fig. 12 ML spectra of the SPC@PDMS film at different conditions.

Supplementary Fig. 13 PL spectra of the BPC@PDMS sample at different stages.

Supplementary Fig. 14 Variation in the triboelectric charge (in nC) between the acrylic and *M*P*X*@PDMS (*M* = Ca, Sr, Ba; *X* = Cl).

Supplementary Fig. 15 The correlation of the triboelectric transfer $q_{P\text{-}M}$ and impact ML intensity affected by different mechanical action frequency from 0.25 to 2 Hz.

Supplementary Fig. 16 The correlation between the triboelectric transfer $q_{P\text{-}M}$ and impact ML intensity affected by different surface roughness.

Supplementary Fig. 17 Crystal structural analysis of the *M*P*X*:Eu_x phosphors with different concentrations *x* of dopant Eu from 0.01 to 0.14 (i.e. the ratio of Eu to *M* from 0.2% to 2.8%)

Supplementary Fig. 18 PL spectra of the synthesized apatite phosphors.

Supplementary Fig. 19 Schematic diagram of the preparation process of the inorganic-organic composite film.

Supplementary Fig. 20 Characterization of the ML performance of the inorganic-organic composites by using a near-field ML imaging test system.

Supplementary Video 1 Demonstration of flexibility and dynamic color change of BPC@PDMS under thousands of stretches

Supplementary Video 2 Demonstration of flexibility and dynamic color change of CPC@PDMS under thousands of stretches

Supplementary References

***Note:** Two supplementary videos and one source data file are given as separated documents.

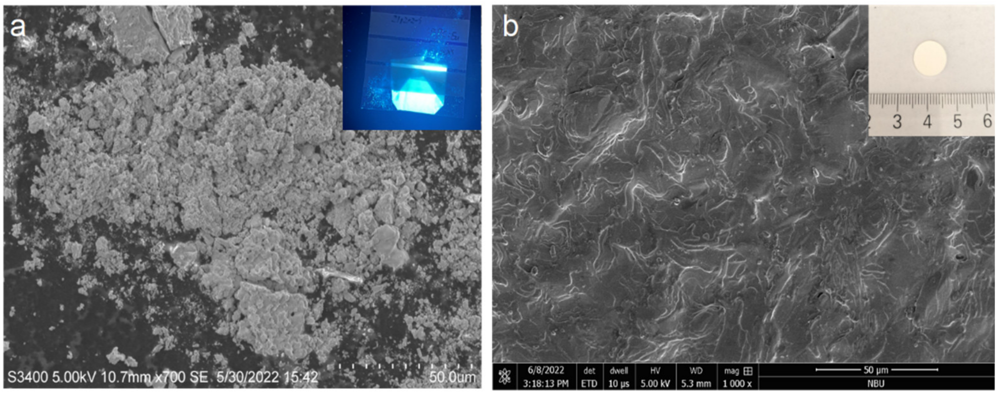

Supplementary Fig. 1: Scanning electronic microscopy (SEM) images and photographs of the BPC phosphors and ceramics. (a) SEM image of the BPC phosphors. The inset is a photographic image of the BPC phosphors being excited by ultraviolet light (365 nm). (b) SEM image of the surface part of the BPC ceramics. The inset is a photographic image of the BPC ceramics under natural light. Other samples (both of phosphors and ceramics) showed the similar morphology as BPC.

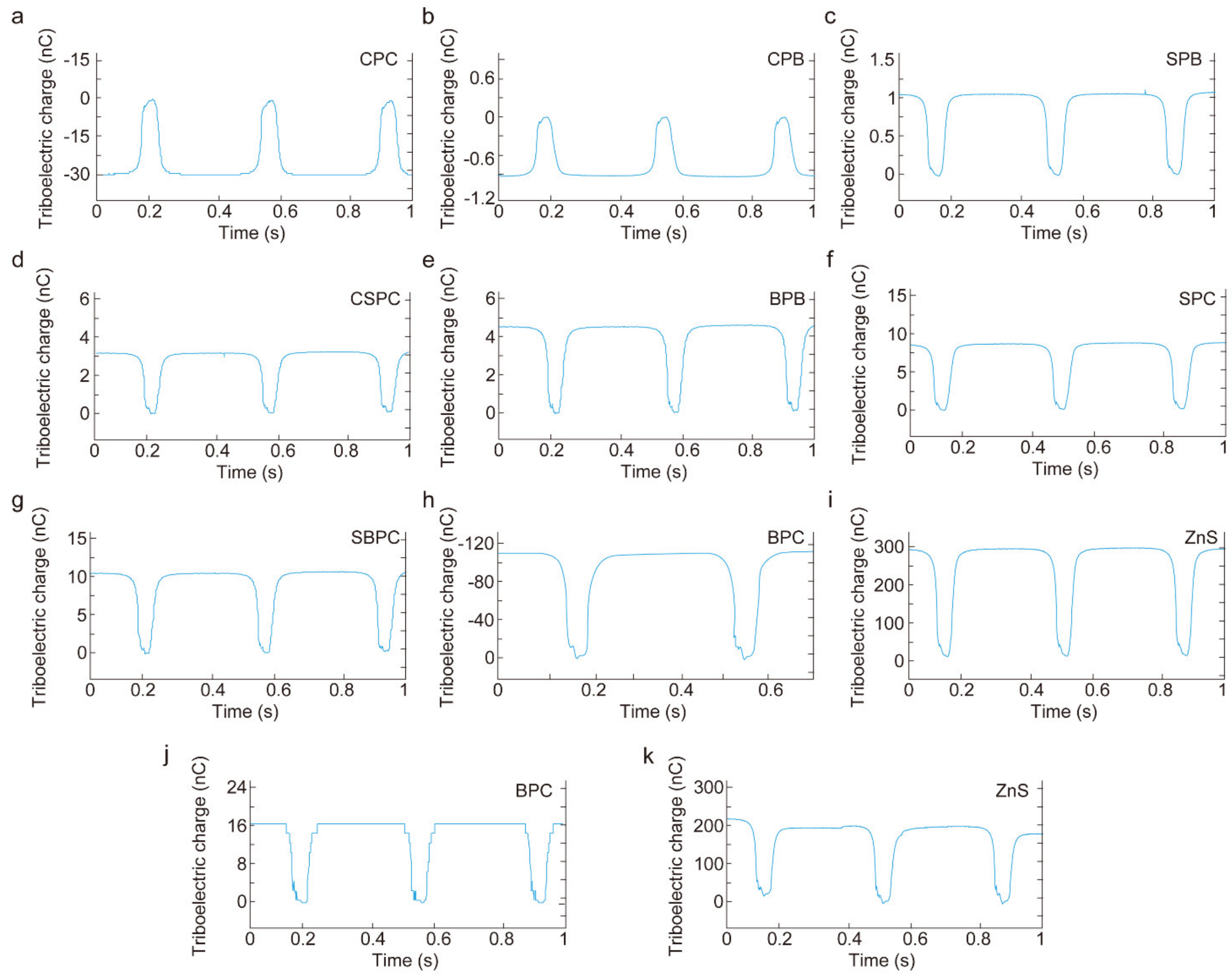

Supplementary Fig. 2: Variation in the triboelectric charge (in nC) between different ceramics and organic film[1]. (a-i) Variation in the triboelectric charge between different ceramics and PDMS in repeated pressing-releasing cycles. (j-k) Variation in the triboelectric charge between different ceramics and silicone (SC) in repeated stretching-releasing cycles. The compositions of the ceramics are denoted at the upper right corner in each figure.

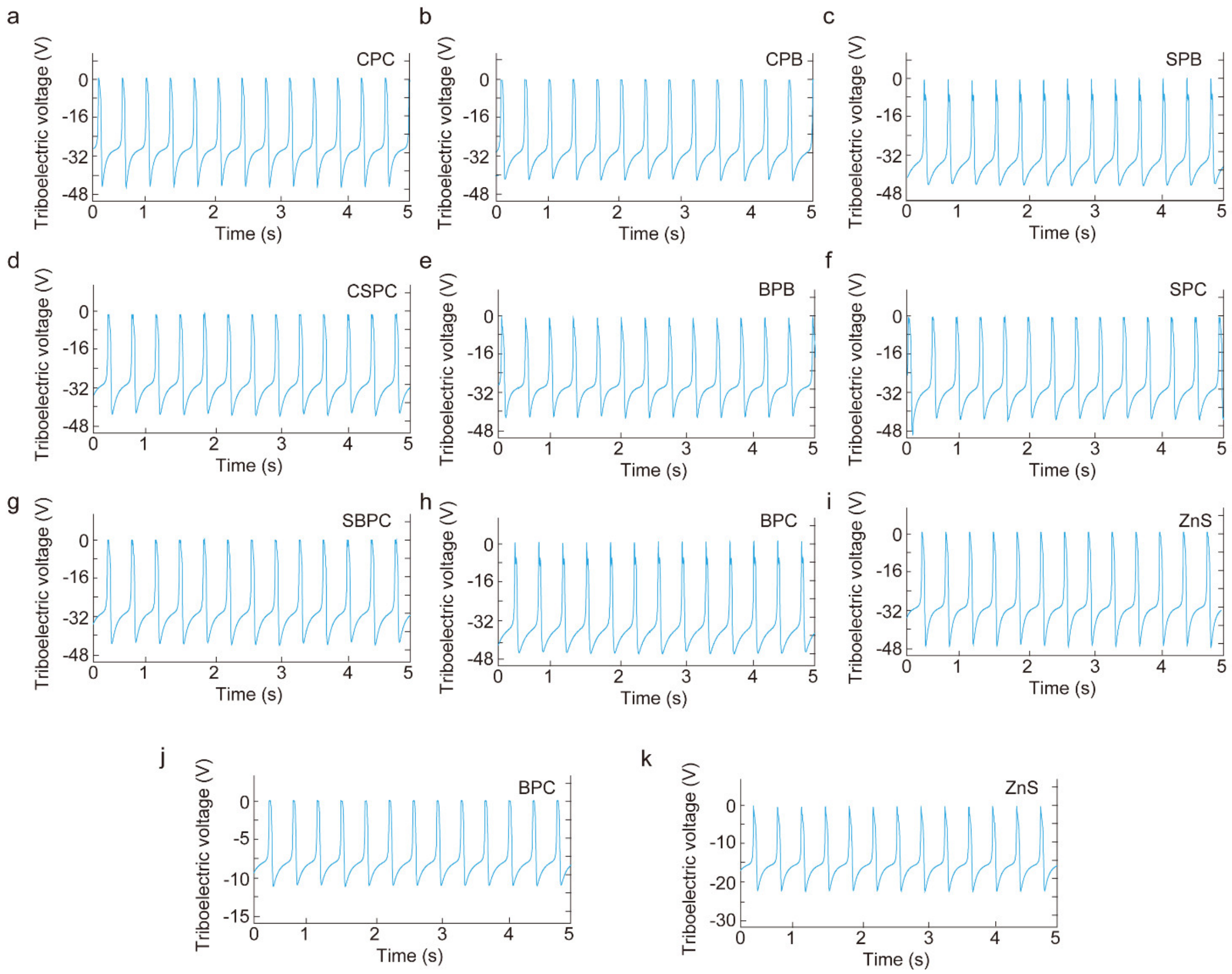

Supplementary Fig. 3: Variation in the voltage (in V) between different ceramics and organic film[2]. (a-i) Variation in voltage between different ceramics and PDMS in repeated pressing-releasing cycles. (j-k) Variation in voltage between different ceramics and SC in repeated stretching-releasing cycles. The compositions of the ceramics are denoted at the upper right corner in each figure.

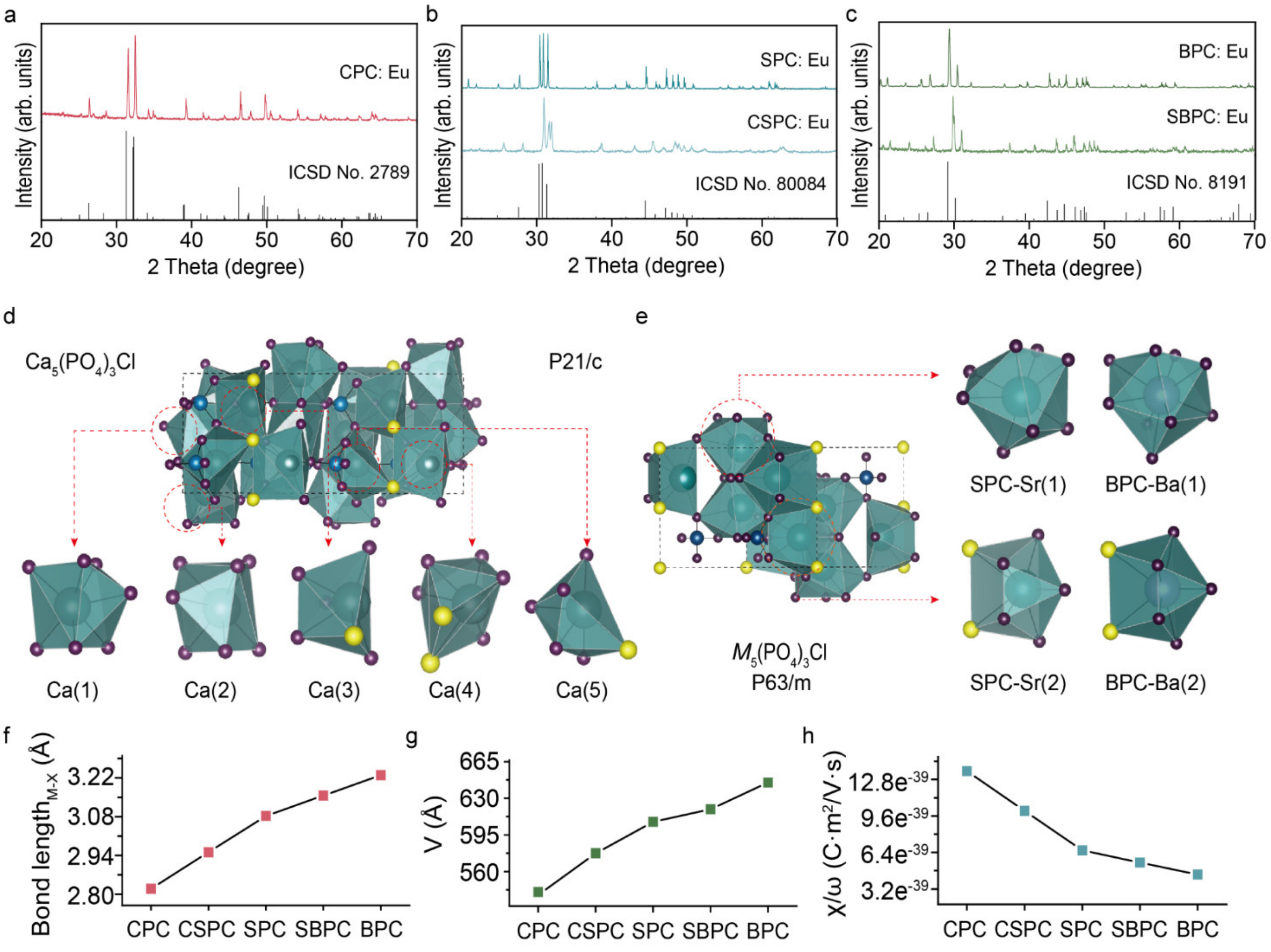

Supplementary Fig. 4: Crystal structures of the apatite phosphors *MPX* (*M* = Ca/Sr/Ba, *X* = Cl)[3]. (a-c) X-ray diffraction patterns and corresponding reference diffraction patterns of CPC [a, inorganic crystal structure database (ICSD) #2789], CSPC and SPC (b, ICSD #80084), SBPC and BPC (c, ICSD #8191). (d-e) Crystal structures of non-piezoelectric hexagonal crystalline structures. The $Ca_5(PO_4)_3Cl$ shows a space group of P21/c (d) and the space group of $Sr_5(PO_4)_3Cl$ and $Ba_5(PO_4)_3Cl$ is P63m (e). *M* sites can be substituted with Eu ions. (f-h) The *M-X* average bond length (f), the single cell volume V (g), and the crystal polarization rate χ to approximate the polarizable properties of crystals (h) calculated from crystal parameters. Here, ω represents the Einstein oscillator[4].

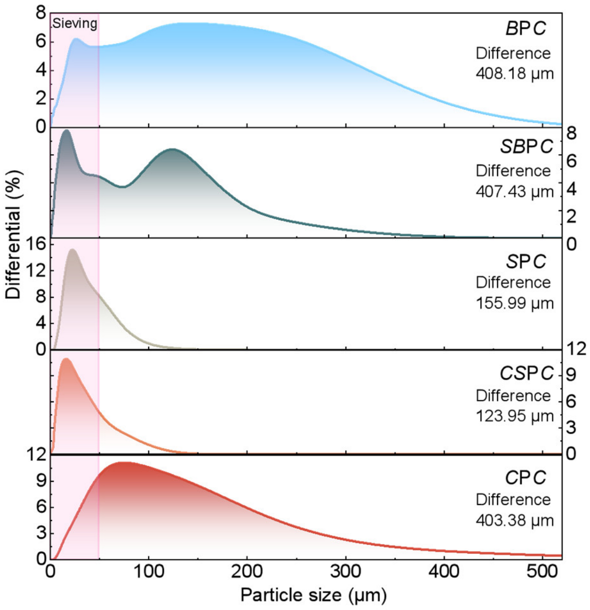

Supplementary Fig. 5: Distribution of particle size of the *M*P*X* (*M* = Ca/Sr/Ba, *X* = Cl) phosphors after sintering. The phosphors were sieved with a 200-mesh sieve and thus the final particle size should be less than 50 μm. The Differential quantifies the proportion of particles within specific size intervals relative to the total number of particles measured, thereby providing insight into the relative abundance of particles across different size ranges within the sample. The differential distribution offers a detailed view of the particle size uniformity and the effectiveness of the sieving process in achieving the desired particle size range for these phosphors.

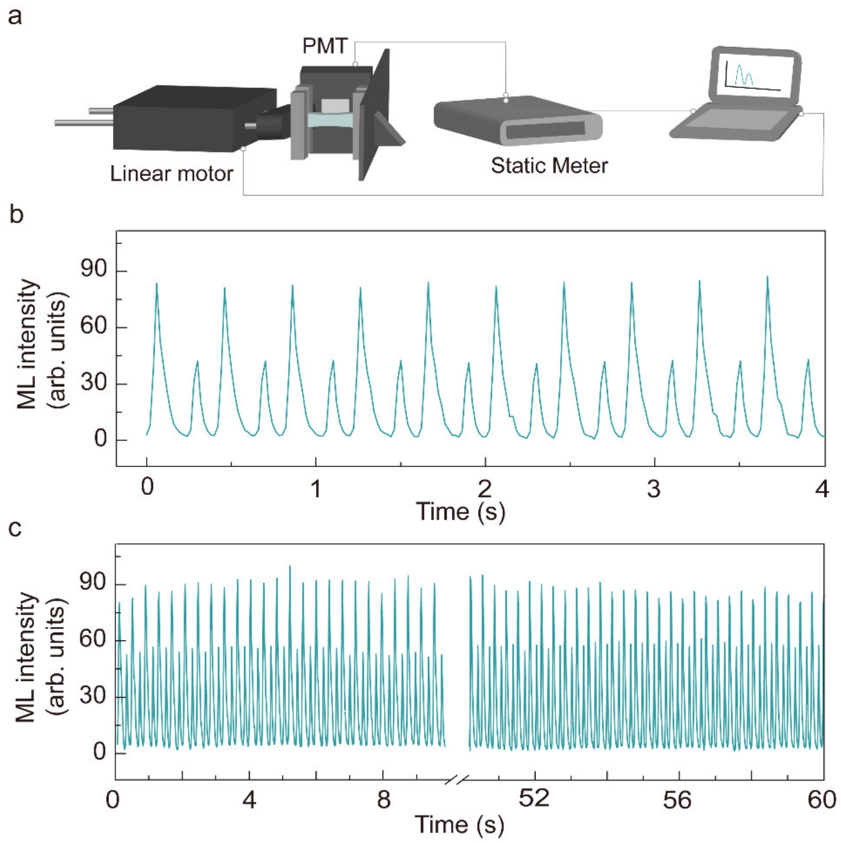

Supplementary Fig. 6: Time-resolved ML measurements of samples upon mechanical stimulus. (a) Schematics of the test device by using a fast-response PMT as the photodetector. (b) Time-resolved ML intensity curve of BPC@PDMS upon repeated stretching-releasing cycles for 60 s. This figure shows the results of the first 10 s (0-10 s) and the last 10 s (50-60 s). The repeated frequency of stretching-releasing cycles is 2.5 Hz. The maximum tensile strain is 20 %. The data acquisition frequency of the static meter is set as 50 Hz. (c) The enlarged part of the first 10 cycles (0-4 s). Two intensity peaks are present in each cycle.

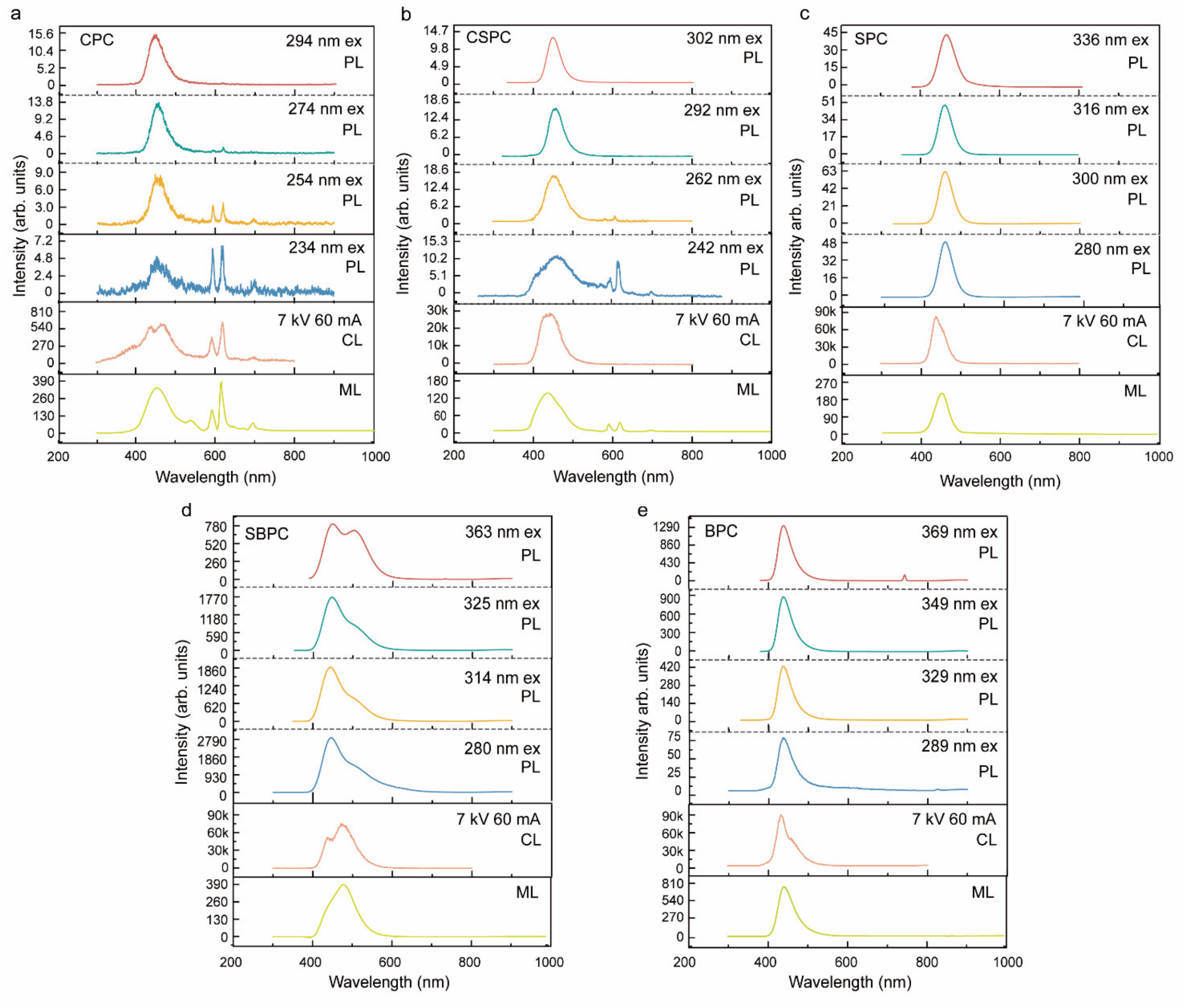

Supplementary Fig. 7: PL, CL and ML spectra of *MPX* phosphors (*M* = Ca, Sr, Ba; *X* = Cl). (a-e) Comparison of emission spectra in each phosphor. The PL spectra of the samples were recorded under excitation of different wavelengths (labelled in the upper right corner). The PL spectra were measured with a multifunctional fluorescence spectrometer (FLS980, Edinburgh Instruments). The CL spectra were recorded by using a modified Mp-Micro-S instrument attached to the SEM (MonoCL4, Gatan). The ML spectra were recorded by using a fiber spectrometer covering the wavelength ranges of 300-1100 nm (QE Pro, Ocean Optics) under mechanical actions (e.g., pressing-releasing cycles). It should be noted that the use of the CCD-type fiber spectrometer with a large slit of results in the broadening of emission bands in the ML spectra.

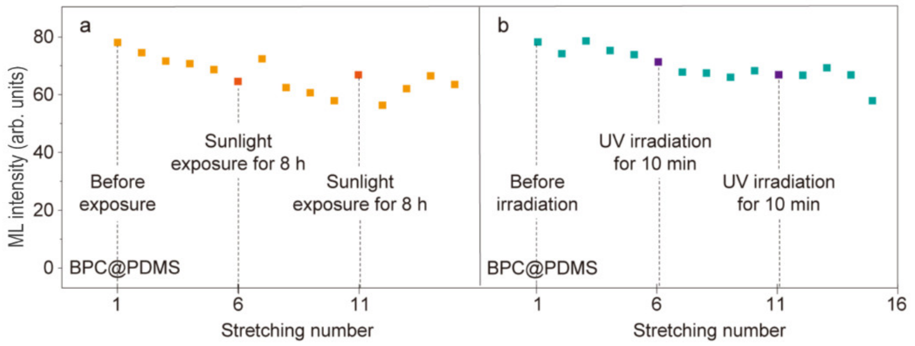

Supplementary Fig. 8: Comparison of the ML intensity in BPC@PDMS with and without light exposure. (a) shows ML intensity without light pre-excitation in the first group of 5 tests (1-5) and the ML intensity with sunlight exposure for 8 h before the second (6-10) and the third groups of tests (11-15), respectively. (b) shows the similar measurements by using UV light (10 min) as the pre-excitation source. The results indicates that pre- excitation of either sunlight or UV light has little influence on the ML intensity. The other samples containing the different apatite phosphors exhibit the similar results.

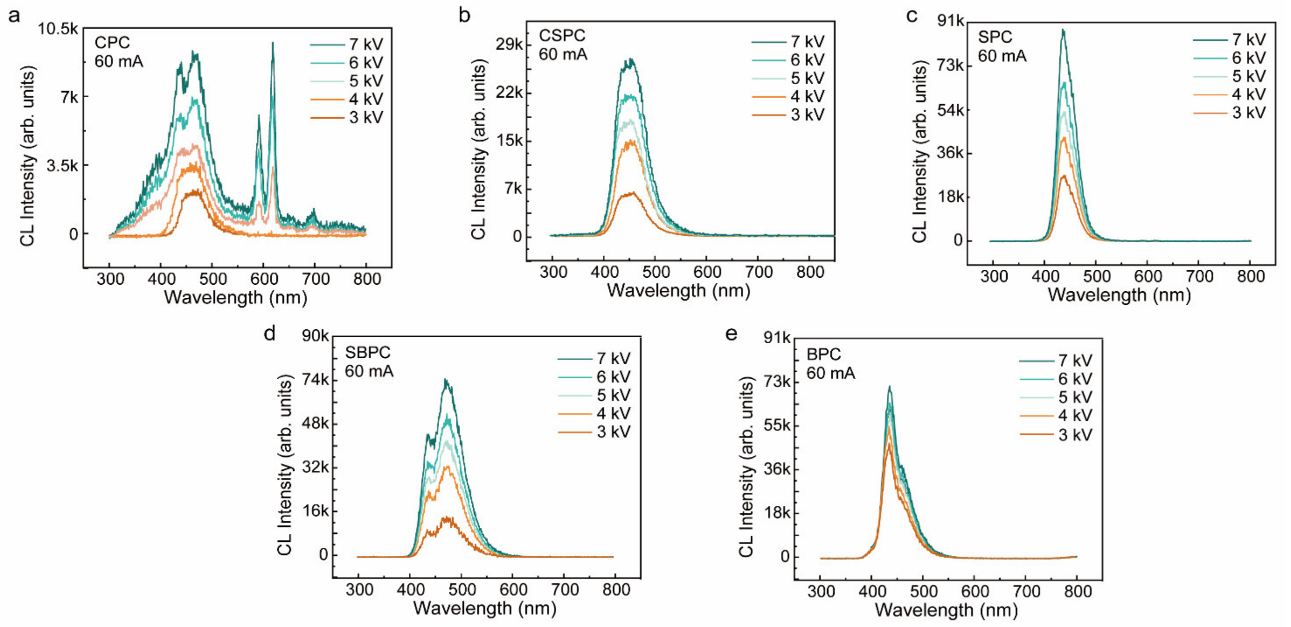

Supplementary Fig. 9: CL spectra of the *MPX* (*M* = Ca, Sr, Ba; *X* = Cl) phosphors by using different accelerating voltages of the electron beam. With the increase of the accelerating volage from 3 to 7 kV, the CL intensity is increased correspondingly. (a-e) are the CL spectra of CPC (a), CSPC (b), SPC (c), SBPC (d) and BPC (e), respectively.

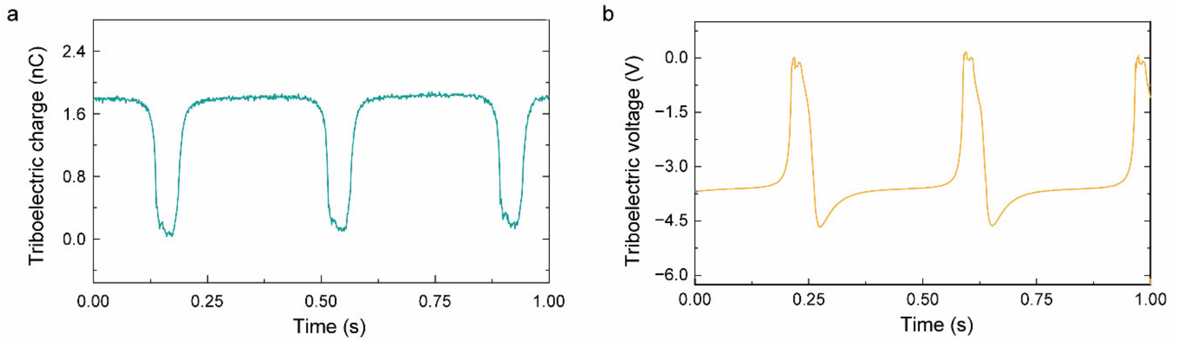

Supplementary Fig. 10: TECD test of a $SrAl_2O_4$:Eu ceramic plate and PDMS film by using the measurement system given in Fig. 1b. (a) Variations in triboelectric charge and (b) changes in voltage between the two materials in pressing-releasing cycles. The repetition frequency of the pressing-releasing cycles was 2.5 Hz. The calculated relative triboelectric series *S* value for $SrAl_2O_4$ is 1.81 according to Eq. (1) and (2).

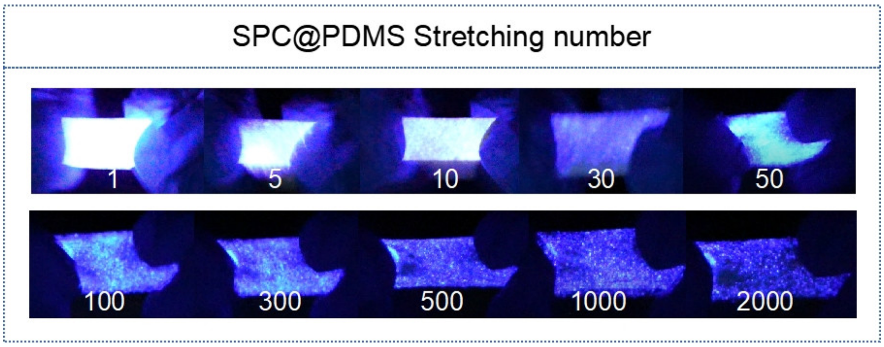

Supplementary Fig. 11: ML photographs of the SPC@PDMS film under stretching for 2000 times. The photographs show variation of ML color due to the self-oxidation effect under the continuous mechanical stimulus. The ML variation indicates valence state conversion of a part of luminescent centers from Eu^{2+} to Eu^{3+}, which is evidenced in the ML spectra as shown in Supplementary Figure 12.

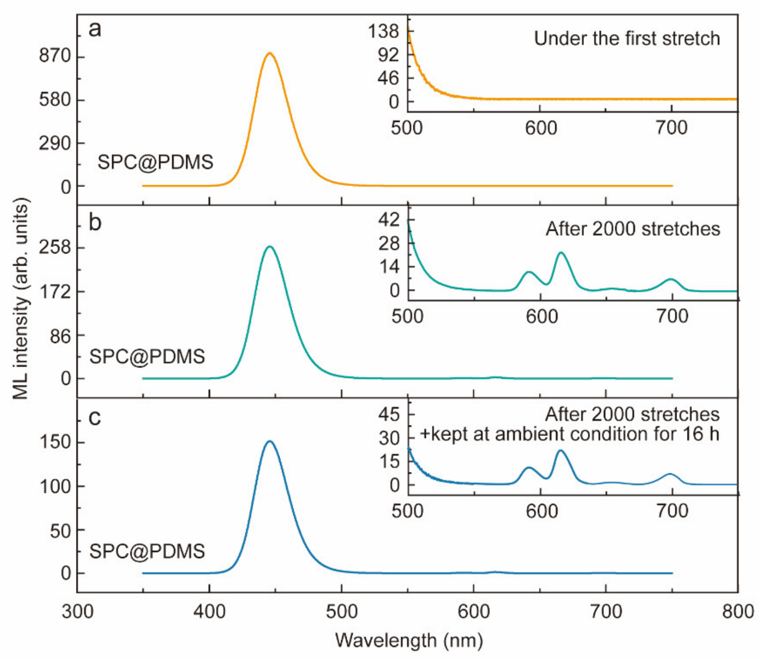

Supplementary Fig. 12: ML spectra of the SPC@PDMS film. (a) under the first stretch, (b) after ~2000 stretches, and (c) further kept at ambient condition for 16 h. The insets show the enlarged part of the spectra focusing on Eu^{3+} emissions. Comparison between (a) and (b) suggests that a small part of Eu^{2+} is oxidized to Eu^{3+} during the continuous stretching. Comparison between (b) and (c) indicates that the valence state change is stable at room temperature.

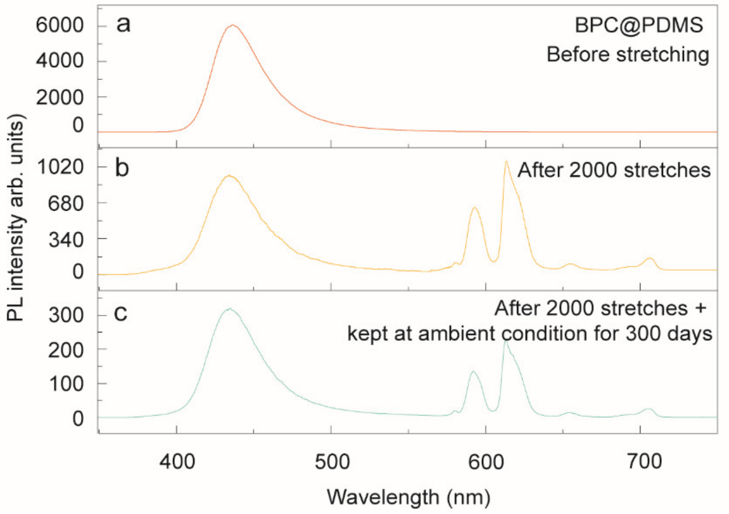

Supplementary Fig. 13: PL spectra of the BPC@PDMS sample at different stages. (a) As-prepared BPC@PDMS composite film without stretching. (b) BPC@PDMS after 2000 stretches. (c) BPC@PDMS after 2000 stretches and then kept at room temperature for one year (~ 300 days). The excitation wavelength is 322 nm.

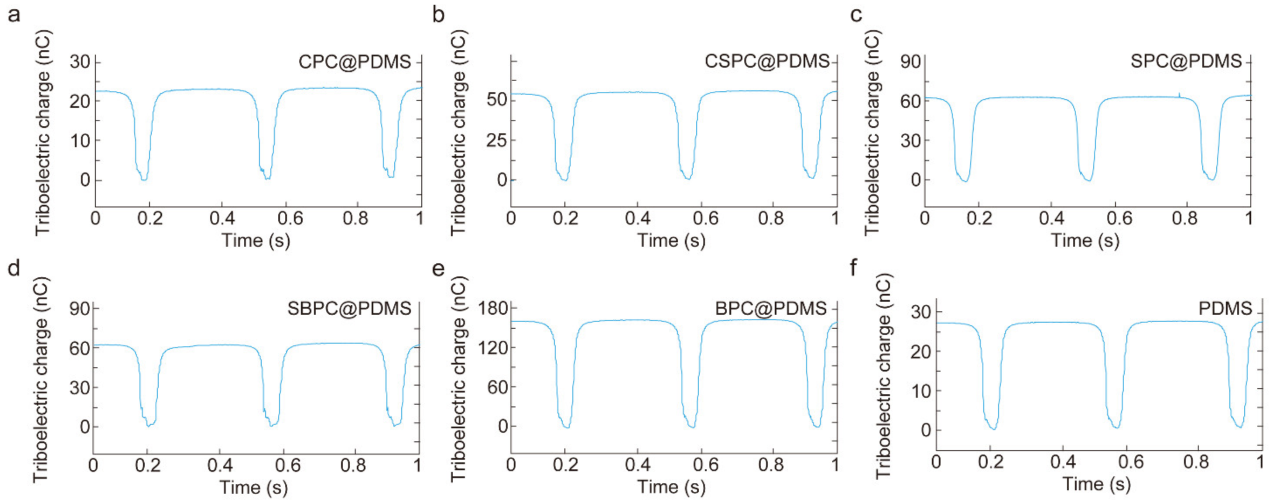

Supplementary Fig. 14: Variation in the triboelectric charge (in nC) between the acrylic and *M*P*X*@PDMS. (a-f) in repeated pressing-releasing cycles[5]. The compositions of the composites are denoted at the upper right corner of each figure.

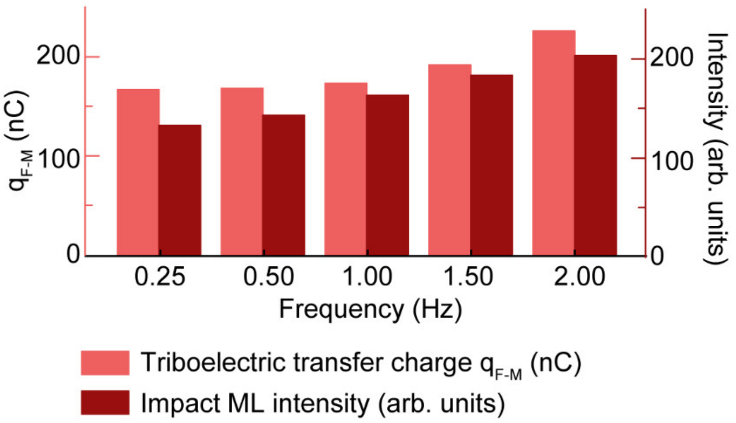

Supplementary Fig. 15: The correlation of the triboelectric transfer $q_{P\text{-}M}$ and impact ML intensity affected by different mechanical action frequency from 0.25 to 2 Hz. The reciprocating distance between the acrylic and *B*PC@PDMS was fixed as ~150 mm. A naturally solidified smooth composite surface was used.

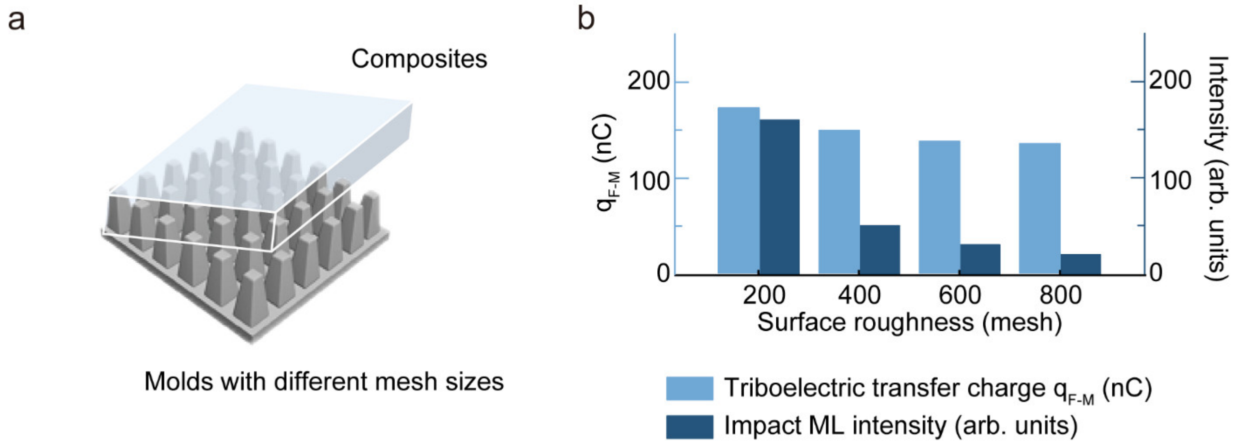

Supplementary Fig. 16: The correlation between the triboelectric transfer $q_{P\text{-}M}$ and impact ML intensity affected by different surface roughness. (a) Schematic diagram of the method for manufacturing composite surfaces with different roughness levels. (b) The correlation between the triboelectric transfer $q_{P\text{-}M}$ and impact ML intensity affected by different surface roughness from 200 to 800 mesh (between acrylic and *B*PC@PDMS, fixed ~150 mm as the reciprocating distance and 2 Hz as the reciprocating frequency).

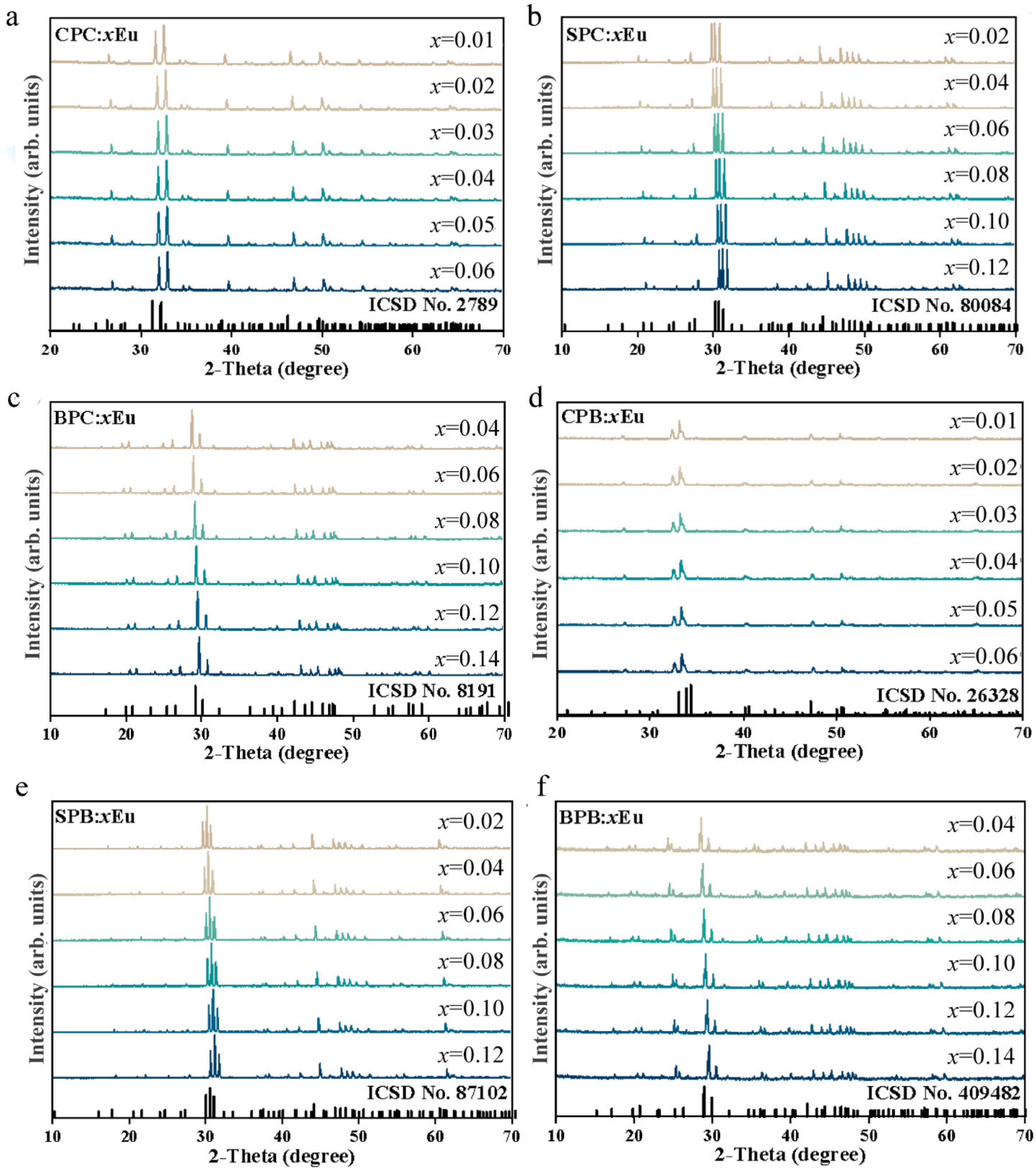

Supplementary Fig. 17: Crystal structural analysis of the *MPX*:Eu_x phosphors with different concentrations *x* of dopant Eu from 0.01 to 0.14 (i.e., the ratio of Eu to *M* from 0.2% to 2.8%)[6]. XRD patterns and the reference diffraction patterns of CPC (a, ICSD #2789), SPC (b, ICSD #80084), BPC (c, ICSD #8191), CPB (d, ICSD #26328), SPB (e, ICSD #87102), and BPC (f, ICSD #409482). In general, the substitution of Eu ions for the *M* sites with a concentration less than 2.8% did not introduce additional impurity phases in all samples.

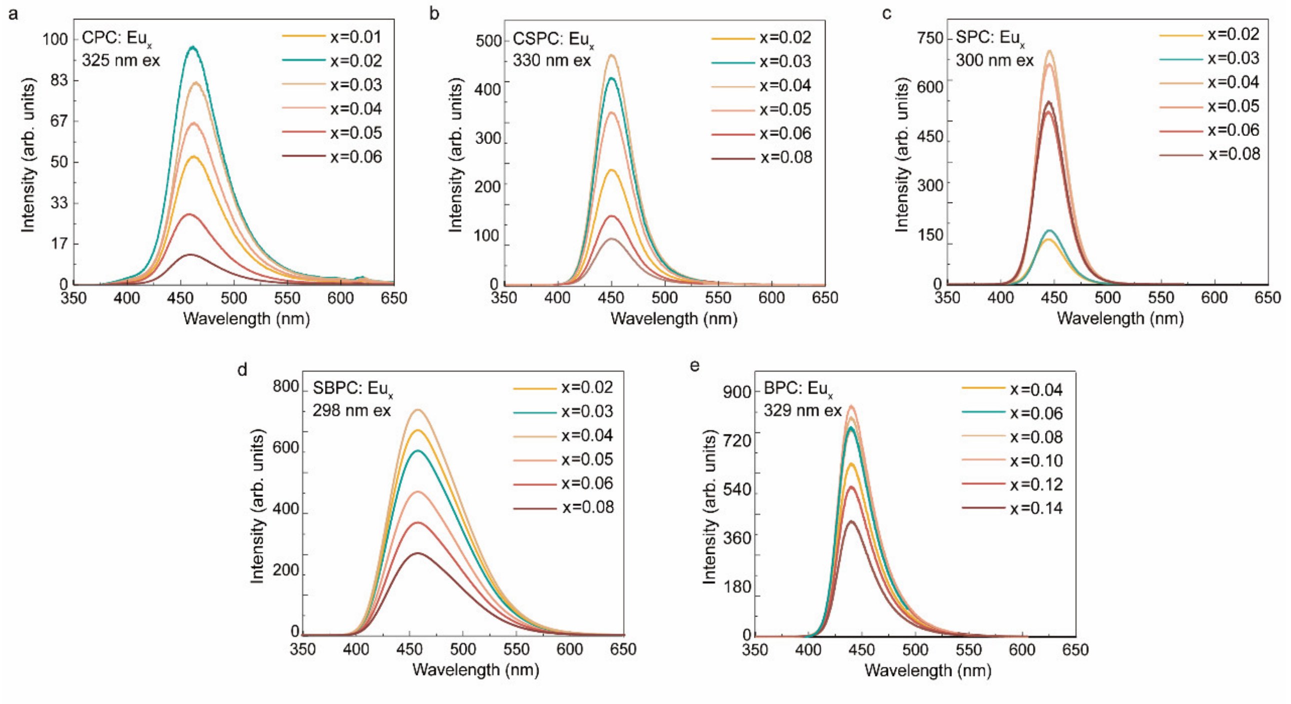

Supplementary Fig. 18: PL spectra of the synthesized apatite phosphors. MPX:Eu$_x$ [M = C (Ca), S (Sr), B (Ba); X = C (Cl), B (Br)] are abbreviations for $M_{5-x}(PO_4)_3X$:Eu$_x$. (a-e) are the PL spectra of CPC (a), CSPC (b), SPC (c), SBPC (d) and BPC (e), respectively. The compositions of the phosphors and the excitation wavelengths are denoted at the upper left corner in each figure. x is the doping concentration of Eu in the phosphors. The sample with the highest PL intensity in each figure is selected from the phosphors for the study of ML-triboelectricity correlation.

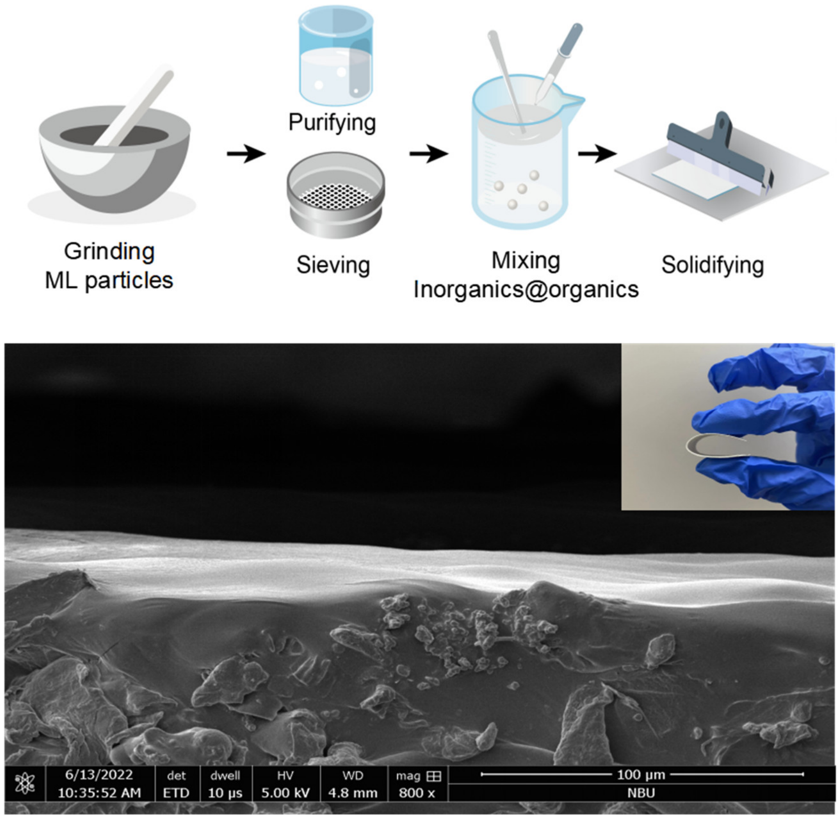

Supplementary Fig. 19: Schematic diagram of the preparation process of the inorganic-organic composite film. The bottom shows the SEM images of the BPC@PDMS film. The inset is a photographic image of the BPC@PDMS film. The film exhibits excellent flexibility.

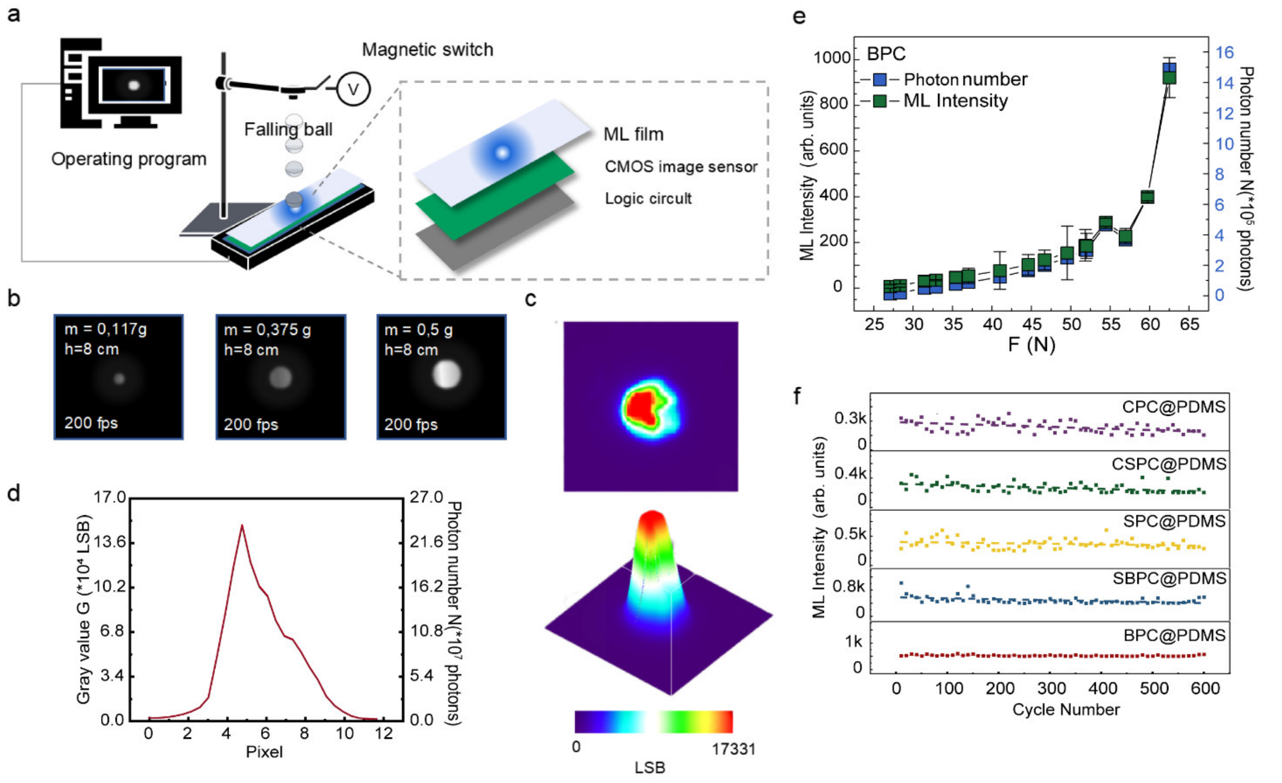

Supplementary Fig. 20: Characterization of the ML performance of the inorganic-organic composites by using a near-field ML imaging test system. (a) Schematic diagram of the near-field ML imaging test system. (b) Variation of grayscale generated by different falling balls impacted onto the BPC@PDMS film. (c) Pseudo-color images of dynamic stress distribution n generated by a falling ball impacted on the BPC@PDMS film. (d) Integrated gray value (left) and integrated number of photons (right) under different pixel. (e) Integrated ML intensity (left) and integrated number of photons (right) under different impact forces from 0.27 to 0.63 N. (f) Variation of ML intensity of various samples under impacted by the same falling ball in 600 tests. The results verified the self-reproducible nature of the ML in the tested materials.

Supplementary references

1. Zou, H., *et al.* Quantifying the triboelectric series. *Nat. Commun.* **10**, 1427 (2019).

2. Zhang, W., *et al.* Measuring the actual voltage of a triboelectric nanogenerator using the non-grounded method. *Nano Energy* **77**, 105108 (2020).

3. Kim, D., Kim, S.-C., Bae, J.-S., Kim, S., Kim, S.-J., Park, J.-C. Eu^{2+}-activated alkaline-earth halophosphates, $M_5(PO_4)_3X:Eu^{2+}$ (M = Ca, Sr, Ba; X = F, Cl, Br) for NUV-LEDs: site-selective crystal field fffect. *Inorg. Chem.* **55**, 8359-8370 (2016).

4. Vorst, A.V., Rosen, A., Kotsuka, Y. *Fundamentals of electromagnetics*. RF/Microwave Interaction with Biological Tissues (2006).

5. Zou, H., *et al.* Quantifying and understanding the triboelectric series of inorganic non-metallic materials. *Nat. Commun.* **11**, 2093 (2020).

6. Wen, J., *et al.* First-principles study on self-activated luminescence and 4f → 5d transitions of Ce^{3+} in $M_5(PO_4)_3X$ (M = Sr, Ba; X = Cl, Br). *Inorg. Chem.* **59**, 5170-5181 (2020).
