# Supplementary material for: Quantifying the interfacial triboelectricity in inorganic-organic composite mechanoluminescent materials
Source: Nat Commun. 2024 Mar 26;15:2673. doi: 10.1038/s41467-024-46900-w (PMC10966096; doi:10.1038/s41467-024-46900-w)
Supplement: Supplementary file 3 — Description of Additional Supplementary Files [file 41467_2024_46900_MOESM3_ESM.pdf]

Description of Additional Supplementary Files:

Supplementary Movie 1: Demonstration of flexibility and dynamic color change of BPC@PDMS under thousands of stretches.

Supplementary Movie 2: Demonstration of flexibility and dynamic color change of CPC@PDMS under thousands of stretches.
